# Sunflower (*Helianthus annuus*) Seed Supplementation in Corn Silage-Based Diets for Dairy Ewes Modifies Milk and Cheese Fatty Acid Profile and Sensory Properties of Cheese

**DOI:** 10.3390/foods14193443

**Published:** 2025-10-08

**Authors:** Manuel Gonzalez-Ronquillo, Beatriz Schettino Bermudez, Jose J. Perez Gonzalez, Alondra Cristel Narvaez Lopez, Lizbeth E. Robles Jimenez, Navid Ghavipanje

**Affiliations:** 1 Departamento de Nutricion Animal, Facultad de MedicinaVeterinaria y Zootecnia, Universidad Autonoma del Estado de Mexico, Instituto Literario 100 Ote, Toluca 50000, State of Mexico, Mexico; mrg@uaemex.mx (M.G.-R.); londycristel1996@gmail.com (A.C.N.L.); 2 Departamento de Producción Agrícola Animal, Universidad Autónoma Metropolitana Xochimilco, Calz. Hueso 1100, Villa Quietud, Coyoacán, Mexico City 04960, Mexico; schettin@correo.xoc.uam.mx (B.S.B.); jjperez@correo.xoc.uam.mx (J.J.P.G.); 3 Instituto de Investigaciones en Ecosistemas y Sustentabilidad, Universidad Nacional Autonoma de Mexico, Antigua Carretera a Pátzcuaro No.8701, Col. Ex Hacienda de Sán José de la Huerta, Morelia 58190, Michoacán, Mexico; 4 Department of Animal Science, Faculty of Agriculture, University of Birjand, Birjand 97175331, Iran; 5 South Khorasan Agricultural and Natural Resources Research and Education Center, Agricultural Research, Education and Extension Organization (AREEO), Birjand 9735197311, Iran

**Keywords:** fatty acids profile, cheese quality, sunflower silage

## Abstract

Consumers increasingly demand dairy products with improved nutritional quality, particularly regarding their fatty acid (FA) composition, due to recognized implications for human health. This study aimed to evaluate the modification in the composition, FA profile, and sensory profile of cheeses elaborated with ewe milk, through the diet inclusion of crushed sunflower (*Helianthus annuus*) seeds and sunflower seed silage in corn silage-based diets. The study was conducted with six East-Friesian ewes in a double 3 × 3 Latin square design, including three 21-day periods. Three diets were based on ad libitum corn silage as follows: control (CTRL, without supplementation), sunflower seeds (SFS, supplemented with 86 g/kg crushed sunflower seeds), and sunflower seed silage (SFSS, supplemented with 137 g/kg sunflower seed silage). The composition and FA profile of milk and cheese, and the sensory properties of cheese, together with the sensory profile, were evaluated. Dietary feeding with SFS and SFSS did not affect milk production and milk fat percentage but increased protein percentage. SFS and/or SFSS increased C18:0, C18:1 trans-9, and C18:1 cis-9 compared to CTRL in milk and cheese. Cheeses from SFS ewes showed improved taste and total acceptability, while odor, color, and texture of cheese remained unaffected. Therefore, SFS and SFSS appeared as a viable strategy to increase the contribution of FA with beneficial effects for health in milk and cheeses.

## 1. Introduction

Livestock production must expand and intensify to meet the rising global food demand while at the same time mitigating environmental impacts, addressing public concerns, and integrating circular economy concepts [1]. Feeding costs account for 70–90% of the variable costs of livestock production in intensive systems and affect productivity, profitability, welfare, and product quality [2]. The post-COVID-19 period saw a surge in global livestock feed demand and prices, despite burdens from food-feed-fuel resource competition, land degradation, import costs, and climate change—factors that affect feed availability and quality [3]. To overcome these, research has pushed to screen for locally available, novel, and eco-friendly feed options that can not only reduce production costs but also boost sustainable practices [1].

Sunflower (*Helianthus annuus*), an oilseed crop from the *Asteraceae* family, is native to North America; however, its adaptability to varied climates and soils has facilitated its cultivation throughout the world, positioning it among the top oilseed crops, alongside soybean, cottonseed, and rapeseed [4]. Currently, the global sunflower seed (SFS) production has reached 54 million metric tons, ranking it as one of the most produced oilseed crops alongside rapeseed, soybean, and cottonseed [5]. Sunflower is suited for short growth cycles (90–120 days) and early sowing potential in some regions like Mexico and the U.S., along with frost resilience [6], rich in C18:2 (65%), 18:1 (22%), and 18:3 (0.4%) fatty acids [7]. Unlike corn cultivation, that associated with high water needs for irrigation, sunflowers are usually cultivated under rainfed practices, positioning them as an eco-friendly alternative when corn cultivation faces environmental challenges [6]. It has been documented [8] that silage prepared from whole sunflower seed offers comparable nutritional value to corn silage, with superior protein and fat content.

Rising consumer awareness of the dairy health benefits has dictated the need for dietary modifications in dairy ruminants to improve milk fatty acid (FA) profiles for human consumption [9,10]. Literature has shown that oilseed processing by-products, such as rapeseed meal and sunflower meal, can serve as alternative protein feeds in ruminant diets and also improve milk FA profiles [8,11,12]. The industrial production of sunflower oil, initiated over a century ago, has prompted extensive research into the use of sunflower by-products in ruminant nutrition over the past four decades [11]. It has been well documented [11] that sunflower seed byproducts contain 92.6% dry matter (DM), 30.2% crude protein (CP), 7.00% ether extract (EE), 42.2% neutral detergent fiber (NDF), 28.80% acid detergent fiber (ADF), and 5.9% ash (as fresh matter). Feeding crushed SFS in dairy cow diets increased milk yield of preformed FA and proportion of unsaturated fatty acids (UFA) [13]. In another study, Vargas-Bello-Pérez et al. [14] reported that dietary supplementation with whole SFS reduced C16:0 levels and increased total C18:1 trans-11 in the milk of Alpine goats. Also, a recent meta-analysis [15] showed that dietary inclusion of sunflower oil in dairy goats enhanced C18:1 cis-9, C18:1 trans-11, C18:2 cis-9 trans-11, C18:3 n-3, and polyunsaturated fatty acids (PUFA) while diminishing saturated fatty acids (SFA) in milk. Despite this, there is still limited information on the impacts of dietary SFS on sheep milk composition and FA profile in the literature, and there remains a gap in the resulting implications for dairy product quality.

Based on the above, this experiment was aimed to evaluate the impact of whole sunflower seed (SFS) and sunflower seed silage (SFSS) on (1) milk production, composition, and FA profile of dairy ewes, and (2) composition, FA profile, and sensory attributes of the resulting cheese. The hypothesis was that dietary SFS and/or SFSS would improve the quality of fat in both milk and cheese by reducing SFA and increasing UFA content. While the effects of sunflower oil supplementation on milk FA profile in small ruminants have been previously reported, research on whole SFS and/or SFSS in ewes is very limited. Moreover, no previous studies have investigated the carry-over effects on both the FA profile and sensory quality of sheep cheese. Therefore, this research will present novel data with practical implications for both academia and industry, looking for sustainable ruminant nutrition strategies that align with consumer preferences.

## 2. Materials and Methods

This article is part of a larger study evaluating the effects of SFS and/or SFSS supplementation on dairy ewe performance, nutrient digestibility, and product quality. Here, we focus specifically on the impact of SFS and/or SFSS on milk and cheese composition, fatty acid profiles, and sensory properties, while detailed data on nutrient intake, digestibility, and lactation performance are reported in the companion paper [8].

### 2.1. Animals, Diets, and Design

All procedures were authorized under the Animal Experimental Guidelines of the Universidad Autonoma del Estado de México (project number UAEMex 4974/2020). A detailed description of the study animals, silage preparation, diets, management practices, and milk production was provided in our companion manuscript [8]. Briefly, six primiparous East-Friesian ewes [70 ± 5 days in milk (DIM), 0.650 ± 0.140 kg/d milk production level; mean ± standard deviation (SD)] were enrolled in a double 3 × 3 Latin square design with three periods of 21 days, consisted of 15 d for diet adaptation and 6 d for sample collection, at the Universidad Autónoma del Estado de México.

Ewes were moved into individual metabolic cages (1.2 m × 0.8 m) in a ventilated enclosed barn and fed with three corn silage-based diets as follows: control (CTRL, without supplementation), sunflower seeds (SFS, supplemented with 86 g/kg crushed sunflower seeds), and sunflower seed silage (SFSS, supplemented with 137 g/kg sunflower seed silage). Diets were formulated to meet NRC [16] nutritional requirements of mid-lactation dairy ewes [theoretically contain 115 g/kg of CP and 10.04 MJ of metabolizable energy (ME)/kg DM]. The chemical composition of individual feedstuffs is presented in Table 1, whereas Table 2 summarizes the ingredient proportions and the overall chemical composition of the experimental diets (CTRL, SFS, and SFSS).

The method for preparing the SFSS is described in a companion manuscript [8]. In short, 50 kg of seeds was mechanically (Molino AZTECA, model 18, Mexico) crushed to pass through a 2 mm screen. The crushed seeds were thoroughly mixed with water at a 1:1 (w/w) ratio and inoculated with 0.001% (v/w) fresh natural inoculant [Pulque, a Mexican fermented beverage rich in lactic acid bacteria (1.5 × 10^8^ CFU/mL), aerobic mesophilic bacteria (1.2 × 10^7^ CFU/mL), and yeast (1.9 × 10^7^ CFU/mL)]. The mixture was thoroughly homogenized, layered into portions, compacted manually to expel air, and packed into 25 kg polyethylene bags (n = 4 per batch). Each bag was placed inside a rigid plastic container (975 mm height × 594 mm Ø; 208 L capacity) with a sealed hard cover to protect against rodents and birds. The silages were stored under anaerobic conditions at ambient temperature (20–25 °C) and fermented for 24 days until their use in the feeding trial.

### 2.2. Milk Sampling and Analysis

Ewes were milked twice daily [at 06:00 and 16:00 h, local time (GMT)] and milk yield was calculated as the sum of morning and evening milking. Fat-corrected milk (FCM) was determined based on 4% fat content using the following formula: FCM (kg/day) = 0.4 × Milk yield (kg/day) + 15 × Fat yield (kg/day) [17].

On the last 6 days of each experimental period, Individual milk samples (100 mL) were collected by using lactometers. Fresh milk was directly used for the chemical analysis, while the remainder was aliquoted and frozen at −20 °C for later analysis. Milk fat, protein, total solids (TS), non-fat solids (NFS), and lactose contents were determined using a MilkoScan FT 6000 (Foss Integrator IMT: Foss Analytics, Hillerød, Denmark). Milk urea nitrogen (MUN) was measured by the micro-Kjeldahl method, and protein content was calculated using a 6.38 conversion factor (Method no. 991.20) [18].

### 2.3. Cheese Manufacture and Analysis

The protocol of García et al. [19] was followed for cheesemaking. Three cheeses were elaborated per treatment and experimental period (n = 9 cheeses per treatment). To analyze the effect of dietary treatments, milk used for cheese manufacturing was not standardized for fat content. Milk was pasteurized at 62.8 °C (±1 °C) for 30 min, followed by cooling to 35 °C (±1 °C). Then, milk was inoculated at 30–32 °C with 0.1 g L^−1^ of starter culture (*Lactococcus lactis* subsp. *cremoris* and *L. lactis* subsp. *lactis*, R-704, Chr. Hansen, Mexico City, Mexico) and coagulated in 50 min using 10 mL L^−1^ of rennet (Cuamix^®^, Chr Hansen, Mexico City, Mexico) diluted 2% in water. After coagulation, the curd was cut, drained, and salted (15 g/kg as fresh matter) [20], and transferred into cheesecloths placed in 250 g perforated molds. The curd was pressed at 0.05 kg force cm^−2^ for 5–6 h at room temperature, then packed and stored at 4 °C for a maximum of 7 days until analysis for dry matter, fat, and total protein (Kjeldahl method; N × 6.38) [18].

### 2.4. Milk and Cheese Fatty Acid Profile

Milk fat extraction and FA derivatization were performed according to Mele et al. [21], while cheese fat was extracted using the procedure described by Pinto et al. [22]. The FA composition of milk and cheese was analyzed using gas chromatography (GC) on a Shimadzu GC2010 gas chromatograph (Shimadzu, Tokyo, Japan) equipped with a flame ionization detector and a Chrompack CP-Sil88 Varian column (100 m × 0.25 mm i.d., 0.20 mm film thickness). The GC conditions were as follows: hydrogen carrier gas flow rate, 1 mL/min; split/spitless injector with a 1:80 split ratio; and temperature program: 40 °C (1 min), 40–173 °C (2 °C/min, 30 min), 173–185 °C (1 °C/min, 5 min), and 185–220 °C (3 °C/min, 19 min). Temperatures of 270 °C and 300 °C were applied to the injector and detector, respectively. Individual FA methyl esters (FAME) were identified by comparison with a 37-component FAME Mix standard (Nu-Chek Prep Inc., Elysian, MN, USA) and a commercial standard mix of C18:1 isomers derived from partially hydrogenated vegetable oil (Supelco, Bellefonte, PA, USA) [23]. The composition of FA was reported as grams per 100 g of total lipids. Peak areas were quantified using Chrome Card software, and the relative percentage of each FA was calculated. The sum of saturated (SFA), monounsaturated (MUFA), and polyunsaturated (PUFA) fatty acids was also calculated.

Cheese nutritional quality was also assessed by the atherogenic index (AI), reflecting the risk of micro coronary and macro coronary diseases:

AI = ((C12:0 + (4 × C14:0) + C16: 0))/MUFA + PUFA

### 2.5. Physicochemical Composition of Cheeses

The physicochemical composition of the cheeses was analyzed at various stages of ripening (including 0, 60, 120, and 180 days). The DM, fat, ash, and CP content were determined according to the methods outlined by AOAC [18]. All compositional analyses were performed in triplicate to ensure accuracy. The color characteristics of the cheeses were evaluated (180 days maturity) using a portable chromometer (CR-400, Konica Minolta, Osaka, Japan), with measurements in terms of the CIELAB color space model of L* (lightness), a* (redness/greenness), and b* (yellowness/blueness) taken. The chroma (C*) value was calculated as (a^2^ + b^2^)0.5, while the hue angle (h*) was h* = tan − 1 (b*/a*) in accordance with the Commission Internationale de l’Eclairage [24] standard.

Shear force was measured on 0, 60, 120, and 180 days of ripening using a Lloyd texture analyzer (Model LRX, Lloyd Instruments, Hampshire, UK) fitted with a Warner–Bratzler shear blade (50 kg compression load cell).

Three cubic cheese samples (20 × 20 × 20 mm^3^) were taken from the central portion of each cheese. The samples were subjected to shear force test using an Instron 5542 machine (Canton, MA, USA) fitted with a 500 N load cell and a knife with a triangular notch. The crosshead was operated at a speed of 100 mm/min, and the maximum force required to cut each specimen was recorded using the Merlin software. The cheese’s pH was determined in duplicate using a portable pH-meter (Crison Instruments, S.A., Alella, Spain) fitted with a spear-type electrode and an automatic temperature compensation probe.

### 2.6. Sensory Evaluation of Cheese

Sensory evaluation of the cheese samples was performed in a designated sensory analysis room within the Department of Animal Nutrition at the Universidad Autonoma del Estado de México, under controlled artificial lighting conditions. Following Mehaia [25], a panel of 50 untrained judges (25 females, 25 males; ages 25–54 years) was recruited from students and staff of the same department. Cheese samples were cut 24 h prior to evaluation, then placed in evaluation containers and refrigerated overnight. The panelists were trained in sensory evaluation procedures and provided with a standardized vocabulary for cheese attributes. To ensure equilibration to room temperature, the samples were removed from refrigeration 1 h prior to evaluation. For sensory assessment, 15 g portions of cheese were served in coded plastic dishes bearing three random digits, allowing for blind evaluation. To minimize carryover effects, panelists were provided with unsalted crackers and water to clean their palates between samples. All attributes were evaluated on a 5-point scale (1 = dislike, 3 = neither like nor dislike, and 5 = like very much) [26], and ratings were averaged to calculate mean scores. The attributes evaluated were depending on the characteristics of texture (roughness, humidity, elasticity, firmness, friability, gumminess, viscosity, crips, adhesion, greasiness), taste (sweet, salty, acidic, alkaline, bitter, fiery, metallic, refreshing, residual teste, overall persistence) and color, this multidimensional approach aimed to create a detailed sensory profile.

### 2.7. Microbiological Analyses of Cheese

Fifty grams of cheese samples were aseptically transferred to stomacher bags, then 200 mL of 0.2% (wt/vol) sodium citrate solution (Merck, Darmstadt, Germany) was added. The mixture was then homogenized in a Laboratory Blender (Seward, London, UK) for 60 s at room temperature. Serial decimal dilutions were prepared by mixing 10 mL of this homogenate with 90 mL of sterile peptone water at 0.1% (wt/vol) according to International Dairy Federation standard 122B [27]. Unless otherwise specified, all media and supplements were sourced from Merck. Total viable counts were determined using casein-peptone, soymeal-peptone (CASO) agar supplemented with 0.6% yeast extract, incubated at 30 °C for 72 h. Mesophilic bacteria were enumerated on de Man, Rogosa, Sharpe (MRS) agar, incubated under anaerobic conditions (Gas-Pack System, BBL, Becton Dickinson, Sparks, MD, USA) at 30 °C for 72 h as described by Renes et al. [28]. The results were expressed as log10 colony-forming unit (CFU) per g (log CFU/g-ml).

### 2.8. Statistical Analyses

Normality of the data was assessed using the Shapiro–Wilk test. FA profile, milk chemical composition, and cheese physical composition and sensory properties were analyzed using the general linear model (GLM) procedure of the SAS software package (Version 9.2, SAS Inst., Inc., Cary, NC, USA), including the effects of ewe, period, and diet in a Latin square design, with period and diet as fixed effects, and ewe as random effect.

FA profile, chemical composition, and microbiology in cheese were further analyzed using the MIXED procedure for repeated measures over time:

y_ijkl_ = μ + D_i_ + A_j(i)_ + P_k_ + T_l_ + D_i_ × T_l_ + ε_ijkl_

where y_ijkl_ is the dependent variable, μ is the overall mean, D_i_ is the fixed effect of diet, A_j(i)_ is the random effect of the jth ewes within the ith diet, P_k_ is the effect of the period (square) k, T_l_ is the fixed effect of sampling time (repeated measure), D_i_ × T_l_ is the interaction effect between diet and time, and εijkl is the residual error. When a significant interaction was found, one-way ANOVA was conducted to compare means for all diet × time combinations.

Microbiological data were analyzed using XLSTAT software. Least square means (LSM) were calculated and compared by one-way ANOVA. Significance was declared at *p* ≤ 0.05, and tendency levels were set at 0.05 < *p* ≤ 0.10.

## 3. Results

### 3.1. Milk

#### 3.1.1. Milk Yield and Composition

There were no differences between diets for milk production (*p* = 0.695), fat corrected milk (FCM, *p* = 0.101), or yields (g/d) of milk components including fat (*p* = 0.541), NFS (*p* = 0.450), protein (*p* = 0.468), lactose (*p* = 0.466), and total solids (*p* = 0.680) (Table 3). However, feeding SFS to dairy ewes resulted in a higher (*p* = 0.466) fat and protein corrected milk (FPCM) compared to both SFSS and CTRL. In addition, the percentage of milk NFS (*p* = 0.027), protein (*p* = 0.043), and lactose (*p* = 0.042) was enhanced with SFSS followed by SFS, and CTRL.

#### 3.1.2. Fatty Acids Profile in Milk

The effect of diets on the FA composition of ewe’s milk is presented in Table 4. Short-chain fatty acids (C6:0 – C14:0) were decreased (*p* < 0.05) in both SFS and SFSS groups, with the exception of C4:0, which was highest in the SFSS group (*p* = 0.024). The levels of C16:1 (*p* = 0.127), C18:3 n-6 (*p* = 0.295), C20:2 (*p* = 0.231), and C20:3 n3 (*p* = 0.899) did not affected by diets. The highest levels of C14:1 (*p* = 0.041), C15:1 (*p* = 0.042), and C16:0 (*p* = 0.045) were found in the CTRL group, followed by SFS and SFSS. In addition, dietary inclusion of both SFS and SFSS increased the concentration of C18:0 (*p* = 0.001), C18:1 trans-9 (*p* = 0.003), and C18:1 cis-9 (*p* = 0.009) compared to CTRL. However, the highest levels of C18:2 n6 (*p* = 0.001) and C18:3 n3 (*p* = 0.026) were observed in CTRL, followed by SFSS and SFS, respectively, while C18:3 n6 (*p* = 0.295) did not differ between diets. Both SFSS and SFS had a higher (*p* = 0.001) content of C20:0 compared to the CTRL group. SFA concentrations were lowest (*p* = 0.036) in SFSS, followed by SFS and CTRL. Conversely, both SFSS and SFS exhibited higher MUFA (*p* = 0.009) and UFA (*p* = 0.015), while showing lower levels of n6 (*p* = 0.0003) compared with CTRL. The lowest AI was for the SFSS group, followed by SFS (*p* = 0.015).

### 3.2. Cheese

#### 3.2.1. Fatty Acids Profile in Cheese

Feeding both SFS and SFSS diets was associated with a lower (*p* < 0.05) content of short- and medium-chain fatty acids (C4:0-C14:0) compared to the CTRL group, except for C4:0, which showed no significant difference (*p* = 0.109) (Table 5). Also, the levels of C15:0 (*p* = 0.001), C16:0 (*p* = 0.001), C16:1 (*p* = 0.009), C17:0 (*p* = 0.001), and C17:1 (*p* = 0.001) were lower in both SFS and SFSS than in the CTRL group. The highest concentration of C18:0 (*p* = 0.001), C18:1 cis-9 (*p* = 0.001), and C18:1 trans-9 (*p* = 0.001) was for SFSS followed by SFS. The content of C18:3 n6 (*p* = 0.112) and C18:3 n6 (*p* = 0.586) remained unchanged by diets. In addition, the highest concentration of C20:0 (*p* = 0.001) and C20:2 (*p* = 0.001) was for the SFSS group. While PUFA levels remained unaffected by diets (*p* = 0.782), feeding both SFS and/or SFSS resulted in lower SFA (*p* = 0.001) and higher MUFA (*p* = 0.001) and UFA (*p* = 0.001). The lowest n6/n3 ratio was for SFSS, followed by SFS (*p* = 0.031). The AI was also decreased with both SFS and SFSS groups (*p* = 0.001). There were no changes in the FA profile of cheese during ripening and storage (i.e., 0, 60, 20, and 180 days), except for C18:2 n6c (LA) that decreased (*p* = 0.034) throughout ripening and C20:3 n3 that showed the lowest (*p* = 0.004) level after 60 days of ripening.

#### 3.2.2. Cheese Physicochemical Composition of Cheese

Dietary inclusion of SFS and/or SFSS was not associated with significant changes in the content of DM (*p* = 0.533), moisture (*p* = 0.533), fat (*p* = 0.177), and CP (*p* = 0.069) of cheese (Table 6). However, the content of DM and ash increased (*p* = 0.001) during ripening time, while moisture decreased (*p* = 0.001).

Cheese pH decreased in SFSS (*p* = 0.004) (Table 7). Both the SFS and SFSS groups had lower shear force values than the CTRL group (*p* = 0.001). Color assessment of cheese demonstrated that a* (redness, *p* = 0.167) and b* (yellowness, *p* = 0.139), C* (chroma, *p* = 0.168), and H* (hue angle, *p* = 0.168) were not affected by diets. However, the L* (lightness, *p* = 0.042) of cheese decreased in the SFSS group.

#### 3.2.3. Sensory Attributes of Cheese

The results of the sensory evaluation (Table 8) of cheeses from ewes fed CTRL, SFS, and SFS diets revealed no significant differences for most of the attributes, including elasticity (*p* = 0.113), friability (*p* = 0.571), gumminess (*p* = 0.926), viscosity (*p* = 0.828), sweet (*p* = 0.103), salty (*p* = 0.951), residual taste (*p* = 0.861), and overall persistence (*p* = 0.794). Additionally, the odor (*p* = 0.828), color (*p* = 0.192), and texture (*p* = 0.823) of cheese remained unaffected by diets. However, roughness was reduced (*p* = 0.050) and humidity was enhanced (*p* = 0.050) in both SFS and SFSS groups. The highest (*p* = 0.021) greasiness was found in the SFSS, followed by the SFS and CTRL group. Furthermore, the bitter (*p* = 0.006) and spicy (*p* = 0.019) taste was higher in SFSS compared to both SFS and CTRL. Cheeses made from SFS showed a higher taste (*p* = 0.001) and total acceptability (*p* = 0.045).

#### 3.2.4. Microbiology Evaluation of Cheese

No differences were recorded in the overall Coliforms (*p* = 0.423), Mesophiles (*p* = 0.501), and Fungi (*p* = 0.176) counts between the dietary groups (Table 9). Also, there was no change in microbial profile on cheese during ripening time (*p* > 0.05).

## 4. Discussion

This article is part of a larger study that evaluated the effects of SFS and SFSS feeding on intake and digestibility of nutrients, production performance, and milk and cheese FA profile in dairy ewes. Our companion paper [8] revealed that both SFS and SFSS increased intake and digestibility of fiber components (i.e., NDF and ADF). However, nitrogen balance and milk yield remained unaffected by diets. These results demonstrated that both SFS and SFSS can be used in dairy sheep diets as alternatives to typical protein feedstuffs, such as soybean meal. In the present study, we investigated the effect of the dietary inclusion of SFS and SFSS on the composition and FA profile of milk and cheese in lactating ewes.

### 4.1. Milk Yield and Composition

Dietary feeding with SFS and SFSS did not affect milk yield or milk fat percentage, while increased protein percentage, which is in line with previous studies [14], supports the fact that oilseeds typically do not alter milk yield while inducing variable effects on milk protein levels in dairy ruminants. Similarly, dietary supplementation of sunflower oil (at 1.84% DM) changes neither milk yield nor milk fat concentration of dairy goats [29]. A recent meta-analysis of 10 experiments with dairy goats [15] or 33 experiments with dairy cows [30] also showed that dietary sunflower did not change milk production. However, it should be noted that the overall impact of dietary fat on milk yield is influenced by a complex interplay of factors, including fat source, type, dietary level, and lactation stage [31].

The maintenance of milk production despite dietary lipid supplementation is particularly relevant for commercial dairy operations, as it suggests that the nutritional improvements achieved through both SFS and SFSS feeding could be implemented without adversely affecting production efficiency. In line with our results, it has been shown [14] that the dietary supplementation with SFS (80 g/d) in Alpine goats, included in a forage-to-concentrate ratio of 60:40, did not alter milk fat and protein content. However, in the present study, milk NFS, protein, and lactose content were enhanced with SFSS (137 g/kg of diet DM) followed by SFS (86 g/kg of diet DM), within a forage-to-concentrate ratio of 57:43. These findings indicate that both SFS and SFSS provided sufficient nitrogen for rumen microbial protein synthesis, which was subsequently reflected in the milk composition [8]. The superior performance of SFSS over SFS in enhancing milk protein content may be attributed to the fermentation process during silage preparation, which partially breaks down protein-lipid complexes and makes nutrients more available for microbial utilization [32]. Furthermore, the observed increase in milk protein yield may be attributed to the diet’s potential to induce rumen defaunation of ciliated protozoa, thereby enhancing microbial protein synthesis in the rumen [33].

In the present study, feeding ewes with two types of sunflower seed (i.e., SFS and SFSS) did not change milk fat. In contrast, results of a meta-analysis [30] on oilseed supplementation in dairy cows, however, showed that sunflower largely led to a reduction in milk fat, particularly when oil inclusion exceeded 40–50 g/kg DM, which seems to be related to the amount of FA resulting in a high number of intermediate isomers that inhibit the de novo (synthesized a new) synthesis of FA in milk fat. This discrepancy could be explained not only by the disparate responses to dietary lipids among ruminant species, which are linked to differences in rumen microbial communities [34], but also by the lower inclusion levels in our study compared to those where milk fat depression occurred. Moreover, it is a well-known fact that when the diet contains sufficient amounts of fiber, neither small nor large ruminants exhibit reductions in milk fat production [12,35]. Milk fat content remains relatively unaffected when concentrates comprise ≤60% of the diet [36]. In the present study, the forage-to-concentrate ratio was around 57:47, and both SFS and SFSS diets were associated with high NDF content that likely mitigated the adverse effects of dietary PUFA on rumen cellulolytic bacteria [8,12].

### 4.2. Fatty Acids Profile in Milk and Cheese

Feeding both SFS and SFSS diets was associated with a lower content of short- and medium-chain fatty acids compared to the CTRL group; however, the content of C4:0 (butyric acid) was increased by 5.3% and 14.9% in the SFS and SFSS groups, respectively. This outcome is in agreement with Gómez-Cortés et al. [37], who supplemented lactating Assaf ewes with 6% sunflower oil (on a DM basis) and observed increased C4:0 along with reduced C6:0–C14:0, as well as with Gómez-Cortés et al. [37], who fed Manchega ewes a diet containing 6% and 12% extruded linseed and reported similar shifts in the short- and medium-chain FA profile. Greater ruminal butyrate availability under lipid supplementation may contribute to enhanced mammary uptake and secretion of C4:0, as increased ruminal butyrate supply has been shown to elevate milk fat content in lactating dairy cattle [38]. Importantly, this shift also plays a physiological role in regulating milk fat fluidity; when the content of PUFA in milk increases, SCFA such as C4:0, positioned predominantly at the stereospecific numbering (sn)-3 position of milk triglycerides, help maintain appropriate melting characteristics and ensure secretion stability of milk fat globules [39]. Butyric acid, a short-chain fatty acid, exhibits antimicrobial properties and has been linked to various beneficial biological activities, including anti-diarrheal, antioxidant, anti-carcinogenic, and anti-inflammatory effects [40]. From a human health perspective, butyric acid serves as a primary energy source for colonocytes and plays a crucial role in maintaining gut barrier function and immune homeostasis [41].

In agreement with our results, Lashkari et al. [13] reported that dietary supplementation of SFS decreased de novo synthesized FA in milk fat of dairy cows. Short- and medium-chain fatty acids are synthetized entirely de novo or partially by the mammary gland from acetate and beta-hydroxybutyrate produced in the rumen [42]. The mechanism underlying this reduction involves the competitive suppression of acetyl-CoA carboxylase, the rate-limiting enzyme in fatty acid synthesis, by long-chain FA absorbed from the digestive tract [43]. It has been found that feeding UFA reduces mammary gland de novo FA synthesis by increasing the uptake of dietary and ruminally derived fatty acids, thereby inhibiting lipogenic enzymes [44]. However, Luna et al. [29] found no changes in the levels of FA from C6:0 to C12:0 in goat’s milk following supplementation with different levels of sunflower oil. The discrepancy between studies may be related to differences in the form of sunflower supplementation (oil vs. seeds), inclusion levels, and basal diet composition, which can influence the extent of biohydrogenation in the rumen [45].

The observed decrease in C18:2 n6 in SFS (28% lower) and SFSS (27% lower) diets, consistent with Vargas-Bello-Pérez et al. [14], likely resulted from extensive hydrogenation of this FA. This biohydrogenation process is mediated primarily by Butyrivibrio fibrisolvens and other rumen bacteria that convert linoleic acid to stearic acid through various intermediate products, including conjugated linoleic acid (CLA) and trans FA [43]. Chilliard and Ferlay [42] found that oilseed C18:2 is more prone to hydrogenation. In the present study, supplementation with SFS and/or SFSS reduced the levels of C12:0, C14:0, and C16:0 fatty acids in both milk and cheese. This reduction may be considered beneficial, as these fatty acids are classified as atherogenic and have been associated with an increased risk of cardiovascular disease [46]. The atherogenic potential of these SFA is attributed to their ability to increase low-density lipoprotein (LDL) levels and promote inflammatory processes in vascular tissues [47]. Comparable results were observed in studies utilizing oilseeds in dairy goat nutrition [48].

Our results also indicated that the dietary inclusion of both SFS and SFSS increased the concentration of C18:0 (stearic acid), C18:1 trans-9 (elaidic acid), and C18:1 cis-9 (oleic acid) in both milk and cheese. However, the content of C18:3 n6 and C18:3 n3 in cheese remained unchanged by diet. This is in agreement with a previous meta-analysis [12] showed the dietary addition of vegetable sources (including sunflower) led to an increase in the concentration of C18:1 c-9 and C18:0 in cheese of sheep. Similarly, feeding goats with oilseeds increased CLA levels in cheese, likely as a result of ruminal biohydrogenation, in which CLA is an intermediate product [49]. The production of CLA isomers, particularly c9,t11-CLA (rumenic acid), represents one of the most significant health benefits associated with ruminant-derived dairy products, as these compounds possess anti-carcinogenic, anti-atherosclerotic, and immunomodulatory properties [50].

The high level of C18:0 has been widely reported in products obtained by dairy animals fed with ingredients containing high levels of UFA, testify a microbial biohydrogenation of the dietary MUFA and PUFA [34]. Duodenal flow of C18:0 has been positively correlated with dietary unsaturated FA intake [51], suggesting that the increase in milk and cheese C18:1 c-9 content likely resulted from the conversion of C18:0 to C18:1 c-9 via mammary delta-9 desaturase. Notably, in dairy cows, mammary desaturation of C18:0 to C18:1 c-9 is directly proportional to the uptake of C18:0 by the mammary gland [51]. In the present study, milk and cheese from the SFS and SFSS groups display a more favorable FA profile, characterized by elevated levels of bioactive FA, such as C18:1 c-9, which have been demonstrated to exhibit anti-cancer properties and induce apoptosis in experimental models [52]. These results were also confirmed by Vargas-Bello-Pérez et al. [12]. The anti-cancer mechanisms of C18:1 c-9 include the suppression of oncogene expression, particularly HER2/neu, and the enhancement of the effectiveness of anti-cancer drugs through membrane composition modifications [53].

It has been well established [54] that the ripening time may be one of the factors affecting the concentration of FA in cheese. However, in the present study, no significant changes in the FA profile of cheese were observed during ripening and storage, except for a decrease in C18:2 n-6c levels throughout the ripening process. This decrease may be attributed to oxidative processes or continued enzymatic modifications by cheese microflora during ripening, particularly lactic acid bacteria that possess lipase and esterase activities [55].

A meta-analysis [12] of 14 published articles showed that the dietary supplementation of vegetable sources rich in unsaturated FA, including sunflower, was associated with higher PUFA and a decrease in SFA content in sheep milk and cheese. Previous studies have also demonstrated that milk from both cows [56] and sheep fed sunflower contains a lower concentration of SFA and a higher concentration of MUFA and PUFA. Likewise, a meta-analysis [30] reported that incorporating oilseeds into cow diets decreases milk SFA content and increases UFA levels, primarily by modifying rumen fermentation and reducing the production of acetic acid, the main precursor for de novo synthesis of short- and medium-chain SFA. Propionic acid production can increase due to glycerol release from triglyceride lipolysis and changes in dietary carbohydrate composition, altering the acetic-to-propionic acid ratio, favoring UFA content in milk [30]. The consistency of these findings across different ruminant species suggests fundamental metabolic pathways are governing FA metabolism in response to dietary oilseed supplementation. These include de novo synthesis (synthesized a new) of FA in the mammary gland, desaturation via stearoyl-CoA desaturase, elongation of FA, and modifications in ruminal biohydrogenation of long-chain PUFA [44,45]. However, the magnitude of response may vary depending on species-specific differences in digestive physiology and metabolic capacity [34]. In our study, a favorable outcome is also the fact that AI and n6/n3 were decreased in the SFS and SFSS groups compared to the CTRL, since higher AI and n6/n3 ratios are considered harmful for health [57], which agrees with Klir Šalavardić et al. [49] who reported better AI in cheese from goats fed oilseed. The reduction in AI is particularly significant because it indicates a lower potential for the dairy products to contribute to cardiovascular disease risk, with values below 1.0 being considered favorable for human health [58]. The role of dietary SFA in cardiovascular disease risk remains a topic of debate [52]. Recent evidence suggests that the health effects of saturated fatty acids may depend on their chain length, food matrix, and overall dietary context rather than their absolute content [59]. Furthermore, the improved n-6/n-3 ratio contributes to a better balance of pro-inflammatory and anti-inflammatory eicosanoids, which is crucial for maintaining optimal immune function and reducing chronic disease risk [60]. Overall, our findings suggest that feeding dairy sheep with SFS and/or SFSS may be a viable strategy for producing milk fat with a more favorable FA profile for human health.

### 4.3. Physicochemical and Sensory Properties of Cheese

Dietary inclusion of SFS and/or SFSS did not alter the content of DM, moisture, ash, fat, and protein in cheese. In confirmation, Zhang et al. [61] showed that the dietary feeding of 260 g/kg SFS to lactating ewes was not associated with changes in fat and protein content of cheese. Similarly, cheese composition was not affected by oilseed inclusion in cow diets [62,63]. Also, the protein and ash content of semi-hard cheese were not affected when goats were fed diets enriched with linseed or pumpkin seed [49]. Likewise, the addition of vegetable oils to goat diets did not alter cheese composition [64]. The maintenance of basic cheese composition parameters following SFS and/or SFSS despite altered milk FA profiles indicates that the fundamental protein and moisture relationships in cheese matrix formation remain intact. This is crucial for cheese processing standardization and product consistency [65]. Additionally, in the present study DM of cheese was increased during ripening time, while the moisture decreased. It has been well established that cheese ripening can alter composition by interacting with internal enzymes or producing acidic compounds, which may influence cheese maturity [20]. It has been well established that the compositional changes during ripening are primarily driven by proteolytic and lipolytic activities of both endogenous milk enzymes and microbial enzymes from starter and non-starter lactic acid bacteria [66].

In our study, cheese pH was decreased by 9.8% in SFSS, which may be attributed to the presence of organic acids produced during the silage fermentation process that were subsequently transferred through the milk to the cheese matrix. Cheeses with lower pH values, indicating conditions closer to the isoelectric point of casein, tend to exhibit firmer and gummier textures, while those with higher pH levels generally display a more pliable and plastic consistency [64]. Also, both SFS and SFSS groups had lower shear force, indicating softer cheese. Consistent with our results, de Medeiros et al. [64] showed that dietary oilseed supplementation for goat resulted in softer cheese. The softening effect may be attributed to the increased proportion of UFA, which have lower melting points and contribute to a more fluid lipid phase within the cheese matrix [67]. The change in the texture of the dairy products, mainly butter and cheese, following alteration in milk FA composition is an already known fact [67]. Cheese produced from milk with a lower content of UFA is firmer, less creamy, and elastic [67]. Thus, increasing UFA concentration in both SFS and SFSS partially explains the lower shear force in these groups. In line with our results, a decrease in butter hardness was reported by Oeffner et al. [68] for milk from Holstein cows fed diets supplemented with oilseeds. Dietary supplementation of oilseeds for dairy cows was also associated with a softer texture in Raclette cheese [69].

Color assessment of cheese demonstrated that a* (redness) and b* (yellowness), C* (chroma), and H* (hue angle) were not affected by diets. However, the L* (lightness) of cheese was reduced by 7.8% in the SFSS group. It has been well established [64] that the addition of oilseeds to the diets may have exerted a positive impact on instrumental color parameters a* and b*. The selective effect on cheese color, specifically the reduced lightness (L*) in the SFSS group, suggests the presence of chromophoric compounds derived from the silage fermentation process. Ensiling can lead to the formation of new chromophoric compounds, which are primarily secondary metabolites or fermentation by-products [70]. These include phenolic compounds released from the breakdown of lignin and flavonoids, as well as Maillard reaction products formed between residual sugars and amino acids under the mild conditions of silage fermentation. Such chromophoric compounds can survive the silage process, enter the milk via the diet, and ultimately be incorporated into the cheese matrix [71].

The sensory properties of cheese are linked to various factors, including not only the physicochemical characteristics of milk and the cheesemaking technology but also to the diets fed to animals [72]. The present study revealed that the sensory evaluation of cheeses from ewes fed CTRL, SFS, and SFSS diets was similar in most attributes, except roughness and humidity. The maintenance of most sensory attributes is crucial for consumer acceptance and indicates that the nutritional improvements achieved through sunflower seed supplementation do not compromise the organoleptic quality of the cheese. Cheeses from SFS ewes showed improved taste and total acceptability, while odor, color, and texture remained unaffected. The improved taste in SFS cheese may be related to the production of flavor compounds during the biohydrogenation process or to the presence of naturally occurring antioxidants in sunflower seeds that enhance flavor stability [73]. Similarly, it has been reported [29] that the sensory attributes of the ewe cheeses remained unaffected by diets enriched with sunflower oil. Present results align with those previously reported by Pascual et al. [74], who reported that cheese made from milk obtained from ewes fed oilseed-supplemented diets had acceptable flavor and sensory properties.

### 4.4. Microbiology Evaluation of Cheese

The microbiological analysis of cheese remained unaffected by diets. Similarly, Dokou et al. [63] reported that dietary oilseed supplementation did not affect white cheese microbial count, supporting the conclusion that moderate levels of dietary lipid supplementation are unlikely to disrupt the established microbial balance in cheese. This finding is particularly important as it demonstrates that the dietary modifications did not adversely affect the microbial ecosystem essential for proper cheese ripening and safety [75]. Also, microbial concentration for all cheeses was similar at 180 days of ripening. This finding is consistent with the results of Schlei et al. [76] and Beuvier et al. [75], who reported no significant differences in microbial populations. Temperature fluctuations are a ubiquitous environmental stressor that significantly impacts bacterial growth [76].

The stability of the microbial profile across treatments can be attributed to several factors. First, the pasteurization process (62.8 °C for 30 min) effectively eliminated pathogenic microorganisms while preserving the beneficial starter cultures that are crucial for cheese development [20]. Second, the similar pH values and moisture levels across treatments provided equivalent environments for microbial growth and metabolism [75]. The absence of significant differences in mesophilic bacteria counts is particularly noteworthy, as these organisms are primarily responsible for the primary fermentation of lactose to lactic acid, which establishes the acidic environment necessary for proper cheese ripening. Maintaining a stable microbial population is critical for the shelf life and quality characteristics of cheese [28]. Consistent mesophilic and fungal counts ensure proper lactose fermentation, lactic acid production, and acidification, which are essential for flavor development, texture stability, and prevention of spoilage or off-flavors. The similar mesophilic populations across all treatments indicate that the altered FA profiles did not interfere with the fundamental fermentation processes [28]. Furthermore, these are supported by the stable fungal counts throughout the ripening period, which is crucial for food safety and product quality, as elevated fungal populations could lead to off-flavors, texture defects, or potential mycotoxin production [77]. However, the consistent microbial profile across treatments indicates that the antimicrobial properties of certain FAs present in higher concentrations in treated groups (such as oleic acid and Linoleic acid) did not reach levels that would inhibit beneficial cheese microorganisms [55].

It is worth noting that some FAs, particularly those with medium-chain lengths, possess antimicrobial properties that can influence cheese microbiota [78]. The observed reduction in C12:0 and C14:0 in the SFS and SFSS groups could theoretically affect microbial populations, but our results indicate that these changes were not sufficient to cause measurable alterations in the overall microbiological profile. This suggests that the cheese matrix and ripening environment provide sufficient buffering capacity to maintain microbial stability despite moderate changes in fatty acid composition.

## 5. Conclusions

This study addresses a research gap by evaluating dietary inclusion of SFS and SFSS in dairy ewes, focusing on milk and cheese quality, FA profile, sensory attributes, and microbial stability. Results demonstrate that inclusion of SFS and/or SFSS in corn silage-based diets generally does not adversely affect milk production, fat, or protein content in dairy ewes. Moreover, both SFS and SFSS increased C18:0, C18:1 trans-9, and C18:1 cis-9 compared to CTRL in milk and cheese, suggesting that both may serve as efficient strategies for improving milk FA profiles for human health. Sensory evaluation revealed no compromise in texture, color, or odor, and microbial analysis confirmed that the cheese microbiota and safety were maintained throughout ripening. However, further research, assessing long-term effects on animal health, performance, and product quality, is needed to fully assess the potential benefits of incorporating SFS and/or SFSS for dairy animals, prior to valid conclusions can be drawn about their effectiveness.

## Figures and Tables

**Table 1:** Chemical composition (g/kg DM) of individual feedstuffs used in the formulation of dietary treatments.

Ingredient	Maize Silage	Alfalfa Hay	Sorghum Gain	Triticale Grain	SBM	SFS	SFSS	Mineral Salt ^1^
DM	270	900	932	900	923	927	553	1000
OM	939	900	919	934	905	937	937	-
CP	84	180	80	123	443	166	166	-
Fat	16.8	30	26.7	25	11.9	480	480	-
NDF	545	550	46	231	70	296	296	-
ADF	322	330	23	64	37	195	195	-
ME ^2^, Mj/kg DM	11	10	13	13.2	3.6	17.5	17.5	-

^1^ Multitech de Malta (1.0 kg DM) containing antioxidant 25 mg, calcium carbonate 4.5 g, salt 6 g, ionophore 30 g, zinc oxide 50 g, sodium bicarbonate 6 g, copper sulfate 6 g, ferrous sulfate 20 g, sodium sulfate 125 g, vitamins E18,000 IU, A 3,000,000 UI, D 3, 750,000 IU, potassium chloride 140 g, E.D.D. I ethylene-dynamine 0.500 g, cobalt carbonate 0.090 g, magnesium oxide 500 mg, manganese oxide 36 g, selenium 0.090. ^2^ Calculated from NRC (2007). SBM, Soybean meal; SFS, sunflower seed; SFSS, sunflower seed silage; DM, dry matter; OM, organic matter; CP, crude protein; NDF, neutral detergent fiber; ADF, acid detergent fiber; ^2^ME, metabolizable energy; Extracted data Cardoso-Gutiérrez et al. [8].

**Table 2:** Ingredients proportions and chemical composition (g/kg DM) of the experimental diets.

**Ingredient**	**Forage**		**Concentrate**						
	Maize Silage	Alfalfa Hay	Sorghum Grain	Triticale Grain	Soybean Meal	SFS	SFSS	Mineral Salt	
Control	Ad libitum	339	249	204	165	0	0	43	
SFS	Ad libitum	340	263	102	166	86	0	43	
SFSS	Ad libitum	321	248	96	157	0	137	41	
**Chemical** **composition g/kg DM**	OM	CP	Fat	N	NDF	ADF	ADL	Ash	ME, Mj/kg
Control	932.8	143.4	37.3	22.9	287.0	139.2	66.2	67.2	11.4
SFS	939.1	152.0	45.7	24.3	420.1	239.6	69.4	60.9	11.7
SFSS	940.0	167.3	66	26.8	411.6	234.4	76.8	60.0	11.7

SFS, sunflower seed; SFSS, sunflower seed silage treatment; OM, organic matter; CP, crude protein; N, nitrogen; NDF, neutral detergent fiber; ADF, acid detergent fiber; ADL, acid detergent fiber lignin; ME, metabolizable energy; Extracted data Cardoso-Gutiérrez Et Al. [8].

**Table 3:** Milk yield (kg/d) and composition from dairy sheep’s fed control (CTRL), sunflower seeds (SFS), and sunflower seeds silage (SFSS) treatments (n = 6 per treatment).

Item	Treatment			SEM	*p*-Values		
	CTRL	SFS	SFSS		Diet	Time	D × T
Milk yield, kg/d	0.643	0.789	0.626	0.1452	0.6958	0.6884	0.0038
FCM, %	0.633	0.720	0.527	0.1405	0.1017	0.0001	0.0206
FPCM, %	0.601 ^b^	0.695 ^a^	0.517 ^c^	0.1333	0.0428	0.0002	0.0234
Fat, %	5.78	5.69	4.79	0.4386	0.8103	0.0106	0.0138
NFS, %	8.93 ^b^	9.29 ^ab^	9.72 ^a^	0.2297	0.0275	0.0052	0.2218
Protein, %	4.23 ^b^	4.38 ^ab^	4.58 ^a^	0.1057	0.0438	0.0061	0.5106
Lactose, %	4.00 ^b^	4.15 ^ab^	4.34 ^a^	0.1008	0.0429	0.0059	0.4974
Total solids, %	22.94	23.50	23.43	0.5651	0.7529	0.3442	0.1007
Fat, g/d	40.79	44.19	30.53	9.1289	0.5416	0.0002	0.0114
NFS, g/d	55.90	73.95	61.98	13.4681	0.4502	0.8028	0.0040
Protein, g/d	26.49	34.86	29.09	6.3210	0.4687	0.8266	0.0038
Lactose, g/d	25.05	32.99	27.54	5.9821	0.4667	0.8280	0.0038
Total solids, g/d	148.23	185.99	149.14	34.2486	0.6800	0.6721	0.0028

^a–c^ Mean values within a row followed by different superscript letters were considered significantly different (*p* < 0.05). SEM = standard error of the mean. Fat-corrected milk (FCM 3.5%, kg/d) = [milk (kg/d) × 0.432] + [fat kg/d) × 16.216]. NFS, Non-fat solids.

**Table 4:** Fatty acids (FA, g/100 g Fat) profile in milk from dairy sheep’s fed control (CTRL), sunflower seeds (SFS), and sunflower seeds silage (SFSS) diets (n = 6 per treatment).

Fatty Acid (FA)	Diets			SEM	*p*-Value
	CTRL	SFS	SFSS		
C4:0	3.15 ^b^	3.32 ^ab^	3.62 ^a^	0.088	0.0237
C6:0	2.44 ^a^	2.17 ^b^	2.18 ^b^	0.043	0.0067
C8:0	2.35 ^a^	1.73 ^b^	1.79 ^b^	0.046	0.0001
C10:0	8.11 ^a^	5.95 ^b^	5.65 ^b^	0.121	0.0001
C11:0	0.44 ^a^	0.31 ^b^	0.31 ^b^	0.028	0.0292
C12:0	4.53 ^a^	3.15 ^b^	2.89 ^b^	0.058	0.0001
C14.0	11.52	10.31	9.71	0.426	0.0617
C14:1	0.28 ^a^	0.22 ^ab^	0.21 ^b^	0.017	0.0407
C15:0	1.53	1.17	1.14	0.104	0.0672
C15:1	0.31 ^a^	0.29 ^ab^	0.22 ^b^	0.020	0.0423
C16:0	31.94 ^a^	28.68 ^ab^	26.48 ^b^	1.176	0.0444
C16:1	1.37	0.71	0.94	0.196	0.1271
C17:0	0.76 ^a^	0.45 ^b^	0.50 ^b^	0.049	0.0095
C17:1	0.60 ^a^	0.38 ^b^	0.33 ^b^	0.035	0.0037
C18:0	8.66 ^b^	14.28 ^a^	14.38 ^a^	0.669	0.0014
C18:1 9 trans	0.99 ^b^	1.51 ^a^	1.90 ^a^	0.108	0.0031
C18:1 9cis	17.32 ^b^	23.35 ^a^	25.46 ^a^	1.262	0.0094
C18:2 n6 trans	0.23	0.22	0.26	0.010	0.0954
C18:2 n6 cis	1.96 ^a^	1.40 ^b^	1.43 ^b^	0.052	0.0004
C20:0	0.15 ^c^	0.20 ^a^	0.17 ^b^	0.005	0.0008
C18:3 n6	0.05	0.04	0.05	0.005	0.2949
C18:3 n3	0.33 ^a^	0.17 ^b^	0.24 ^ab^	0.028	0.0262
CLA	0.46 ^ab^	0.36 ^b^	0.53 ^a^	0.029	0.0192
C20:2	0.09	0.15	0.21	0.044	0.2310
C20:3 n3	0.19	0.16	0.18	0.045	0.8991
SCFA	16.05 ^a^	13.17 ^b^	13.24 ^b^	0.264	0.0004
SFA	75.61 ^a^	71.74 ^ab^	68.86 ^b^	1.376	0.0364
PUFA	2.86 ^a^	2.17 ^b^	2.39 ^a^	0.109	0.0111
MUFA	20.60 ^b^	26.25 ^a^	28.86 ^a^	1.271	0.0098
UFA	24.21 ^b^	29.01 ^ab^	31.99 ^a^	1.303	0.0154
n6	2.24 ^a^	1.67 ^b^	1.75 ^b^	0.046	0.0003
n3	0.52	0.34	0.42	0.073	0.2964
n6/n3	4.77	5.25	4.11	0.924	0.6971
PUFA/SFA	0.14 ^a^	0.08 ^b^	0.08 ^b^	0.005	0.0005
AI	3.41 ^a^	2.56 ^ab^	2.13 ^b^	0.201	0.0113

^a,b^ Mean values within a row followed by different superscript letters were considered significantly different (*p* ≤ 0.05). SEM = standard error of the mean. SCFA: Short-chain fatty acids; MCFA: Medium-chain fatty acids; LCFA: Long-chain fatty acids; SFA: Saturated fatty acids; MUFA: Monounsaturated fatty acids; PUFA: Polyunsaturated fatty acids; UFA: Unsaturated fatty acids; CLA: Conjugated linoleic acid; AI: Atherogenic index; n6: Omega-6 fatty acids; n3: Omega-3 fatty acids; SEM: Standard error of the mean.

**Table 5:** Fatty acids (FA, g/100 g fat) profile in cheese from dairy sheep’s fed control (CTRL), sunflower seeds (SFS), and sunflower seeds silage (SFSS) diets (n = 6 per treatment).

Fatty Acids (FA)	Diets			Days				SEM	*p*-Value	
	CTRL	SFS	SFSS	0	60	120	180		Diet	Day
C4:0	3.16	3.26	2.77	3.36	2.77	2.85	3.28	0.165	0.1097	0.0872
C6:0	2.39 ^a^	2.19 ^b^	2.17 ^b^	2.24	2.17	2.30	2.29	0.045	0.0017	0.3267
C8:0	2.25 ^a^	1.72 ^b^	1.72 ^b^	1.93	1.86	1.91	1.89	0.030	0.0001	0.5604
C10:0	8.16 ^a^	5.76 ^b^	5.76 ^b^	6.56	6.56	6.66	6.45	0.084	0.0001	0.5087
C11:0	0.46 ^a^	0.31 ^b^	0.32 ^b^	0.36	0.36	0.37	0.35	0.013	0.0001	0.9037
C12:0	4.56 ^a^	3.09 ^b^	2.93 ^c^	3.51	3.55	3.52	3.53	0.043	0.0001	0.9498
C14:0	11.76 ^a^	9.83 ^b^	9.65 ^b^	10.43	10.60	10.35	10.26	0.138	0.0001	0.4963
C14:1	0.28	0.26	0.22	0.24	0.23	0.23	0.32	0.024	0.2609	0.1006
C15:0	1.63 ^a^	1.20 ^b^	1.17 ^b^	1.31	1.35	1.37	1.30	0.039	0.0001	0.6094
C15:1	0.32 ^a^	0.29 ^a^	0.23 ^b^	0.27	0.28	0.28	0.29	0.009	0.0001	0.7299
C16:0	32.14 ^a^	27.48 ^b^	25.83 ^b^	28.63	29.08	28.00	28.22	0.578	0.0001	0.6764
C16:1	1.23 ^a^	0.97 ^ab^	0.77 ^b^	0.99	0.78	1.12	1.07	0.098	0.0099	0.1789
C17:0	0.72 ^a^	0.53 ^b^	0.52 ^b^	0.57	0.58	0.62	0.56	0.027	0.0001	0.6101
C17:1	0.56 ^a^	0.39 ^b^	0.32 ^c^	0.44	0.41	0.41	0.44	0.013	0.0001	0.4288
C18:0	8.75 ^c^	14.12 ^b^	15.85 ^a^	12.47	12.95	13.59	12.62	0.425	0.0001	0.3969
C18:1 9trans	1.02 ^c^	1.44 ^b^	2.07 ^a^	1.44	1.49	1.67	1.45	0.079	0.0001	0.2592
C18:1 9cis	17.75 ^b^	24.57 ^a^	24.97 ^a^	22.47	22.09	22.40	22.77	0.528	0.0001	0.8898
C18:2n6 trans	0.21 ^b^	0.25 ^a^	0.23 ^ab^	0.24	0.21	0.24	0.23	0.010	0.0395	0.4895
C18:2n6 cis	1.57 ^a^	1.44 ^ab^	1.26 ^b^	1.58 ^a^	1.23 ^b^	1.36 ^ab^	1.51 ^ab^	0.074	0.0197	0.0344
C20:0	0.15 ^b^	0.19 ^a^	0.20 ^a^	0.17	0.19	0.19	0.18	0.005	0.0001	0.1267
C18:3 n6	0.05	0.04	0.05	0.04	0.04	0.05	0.04	0.003	0.1120	0.4294
C18:3 n3	0.23	0.21	0.21	0.25	0.18	0.22	0.21	0.021	0.5866	0.3200
CLA	0.36	0.36	0.44	0.44	0.34	0.39	0.39	0.025	0.0640	0.1182
C20:2	0.09 ^b^	0.14 ^b^	0.24 ^a^	0.16	0.20	0.16	0.12	0.026	0.0013	0.3594
C20:3n3	0.11	0.16	0.17	0.17 a	0.09 ^b^	0.16 ^a^	0.16 ^a^	0.019	0.1322	0.0048
SCFA	15.98 ^a^	12.91 ^b^	12.42 ^b^	14.11	13.32	13.73	13.92	0.22	0.001	0.216
SFA	76.18 ^a^	69.68 ^b^	68.92 ^b^	71.59	72.01	71.78	70.98	0.58	0.001	0.741
PUFA	2.29	2.22	2.17	2.46	1.95	2.19	2.30	0.12	0.782	0.114
MUFA	20.89 ^b^	27.73 ^a^	28.38 ^a^	25.63	25.13	25.89	26.03	0.57	0.001	0.803
UFA	23.83 ^b^	30.58 ^a^	31.23 ^a^	28.77	27.65	28.73	29.04	0.61	0.001	0.572
n6	1.84 ^a^	1.71 ^ab^	1.54 ^b^	1.87 ^a^	1.48 ^b^	1.65 ^ab^	1.79 ^ab^	0.08	0.047	0.041
n3	0.35	0.36	0.37	0.42	0.27	0.37	0.38	0.03	0.901	0.091
n6/n3	6.22 ^a^	4.95 ^ab^	4.36 ^b^	4.70	6.05	4.84	5.11	0.47	0.031	0.366
PUFA/SFA	0.11 ^a^	0.08 ^b^	0.07 ^b^	0.09	0.08	0.08	0.09	0.01	0.001	0.236
AI	3.52 ^a^	2.34 ^b^	2.16 ^b^	2.64	2.81	2.62	2.60	0.09	0.001	0.507

^a–c^ Mean values within a row followed by different superscript letters were considered significantly different (*p* ≤ 0.05). SCFA: Short-chain fatty acids; MCFA: Medium-chain fatty acids; LCFA: Long-chain fatty acids; SFA: Saturated fatty acids; MUFA: Monounsaturated fatty acids; PUFA: Polyunsaturated fatty acids; UFA: Unsaturated fatty acids; CLA: Conjugated linoleic acid; AI: Atherogenic index; n6: Omega-6 fatty acids; n3: Omega-3 fatty acids; SEM: Standard error of the mean.

**Table 6:** Chemical composition (g/100 g) of cheese from dairy sheep’s fed control (CTRL), sunflower seeds (SFS), and sunflower seeds silage (SFSS) diets (n = 6 per treatment).

Item	Diets			Time				SEM	*p*-Value		
	CTRL	SFS	SFSS	0	60	120	180		Diet	Time	D × T
Composition											
DM	78.97	77.44	77.69	58.26 ^b^	85.73 ^a^	85.31 ^a^	82.83 ^a^	1.021	0.533	0.001	0.083
Moisture	21.03	22.55	22.31	41.73 ^a^	14.27 ^b^	14.69 ^b^	17.16 ^b^	1.021	0.533	0.001	0.082
Ash	9.27 ^a^	7.56 ^b^	6.42 ^c^	7.52 ^c^	7.67 ^bc^	7.83 ^ab^	7.99 ^a^	0.081	0.001	0.001	0.001
Fat	42.91	45.97	44.29	45.07	43.13	44.32	45.05	1.125	0.177	0.690	0.035
CP	33.07	29.82	33.07	27.00 ^b^	33.17 ^a^	36.20 ^a^	32.50 ^a^	1.424	0.0699	0.009	0.004

^a–c^ Mean values within a row followed by different superscript letters were considered significantly different (*p* ≤ 0.05). SEM = standard error of the mean. DM, Dry matter expressed as fresh matter.

**Table 7:** Physical composition of cheese from dairy sheep’s fed control (CTRL), sunflower seeds (SFS), and sunflower seeds silage (SFSS) diets (n = 6 per treatment) at 180 d.

Item	Diets			SEM	*p*-Value
	CTRL	SFS	SFSS		
pH	5.31 ^a^	5.23 ^a^	4.79 ^b^	0.074	0.0004
Shear force	3.99 ^a^	2.19 ^b^	1.85 ^b^	0.189	0.0001
Color					
L*	73.26 ^a^	72.50 ^a^	67.50 ^b^	1.677	0.0425
a*	−0.39	−1.73	−0.24	0.599	0.1678
b*	22.24	20.18	20.53	0.764	0.1397
C*	21.82	20.36	20.69	0.555	0.1688
H	91.10	94.97	90.98	1.658	0.1689

^a,b^ Mean values for each experiment within a row with unlike superscript letters were significantly different (*p* ≤ 0.05). SEM = standard error of the mean. L* = lightness (greater values show lighter color), a* = redness (greater values show redder color), C* = chroma or saturation (calculated as (a*^2^ + b*^2^)^1/2^ (greater values display greater total color/more vivid color), b* = yellowness (greater values show more yellow color), h = hue angle (calculated as tan^−1^ (b*/a*), greater values display more shift from red to yellow).

**Table 8:** Sensory properties in cheese from dairy sheep’s fed control (CTRL), sunflower seeds (SFS), and sunflower seeds silage (SFSS) diets (n = 6 per treatment).

Item	Diets			SEM	*p*-Value
	CTRL	SFS	SFSS		
**Texture Characteristics**					
Roughness	3.51 ^a^	2.78 ^b^	3.03 ^b^	0.250	0.050
Humidity (Presence of free serum)	1.86 ^b^	2.54 ^a^	2.58 ^a^	0.174	0.050
Elasticity (semi-soft cheeses)	1.57	1.92	1.83	0.195	0.113
Firmness	4.14 ^a^	3.73 ^a^	3.06 ^b^	0.560	0.001
Friability (very dry cheeses tend to crumble)	2.63	2.43	2.72	0.349	0.571
Gumminess	2.46	2.38	2.19	0.549	0.926
Viscosity	1.51	1.41	1.64	0.257	0.828
Crisp	1.68	1.52	1.53	0.214	0.089
Adhesion (low-moisture cheeses)	1.8 ^a^	1.51 ^b^	2.00 ^a^	0.226	0.001
Greasiness	1.97 ^b^	2.51 ^c^	2.97 ^a^	0.152	0.021
**Taste Characteristics**					
Sweet	1.4	1.62	1.28	0.429	0.103
Salty	2.89	2.84	2.67	0.233	0.956
Acidic	2.31 ^b^	2.19 ^b^	2.97 ^a^	0.213	0.001
Alkaline	1.94	1.97	1.75	0.273	0.861
Bitter	2.40 ^b^	2.22 ^b^	2.92 ^a^	0.250	0.006
Spicy	1.54 ^b^	1.68 ^b^	2.08 ^a^	0.235	0.019
Astringent	1.77	1.84	2.03	0.325	0.909
Acre	2.09 ^a^	1.44 ^b^	2.22 ^a^	0.219	0.048
Fiery	1.63 ^ab^	1.41 ^b^	1.75 ^a^	0.172	0.024
Metallic	1.43	1.49	1.58	0.205	0.274
Refreshing	1.46 ^c^	1.70 ^a^	1.50 ^b^	0.034	0.031
Residual taste	2.60	2.59	2.86	0.271	0.512
Overall persistence	2.37	2.62	2.83	0.468	0.794
Odor	3.04	3.11	3.11	0.203	0.818
**Color**	3.73	3.44	3.2	0.379	0.192
**General Texture**	3.44	3.49	3.00	0.579	0.823
**General Taste**	3.09 ^ab^	3.29 ^a^	2.79 ^b^	0.293	0.001
**Total acceptability**	3.28 ^b^	3.30 ^a^	2.99 ^ab^	0.100	0.045

^a–c^ Mean values for each experiment within a row with unlike superscript letters were significantly different (*p* ≤ 0.05). SEM = standard error of the mean.

**Table 9:** Microbiology in cheese from dairy sheep’s fed control (CTRL), sunflower seeds (SFS), and sunflower seeds silage (SFSS) diets (n = 6 per treatment) expressed as log_10_ CFU/g.

Microbial Groups	Diets			Ripening Time				SEM	*p*-Value		
	CTRL	SFS	SFSS	0	60	120	180		Diet	Time	D × T
Coliforms	0.011	0.290	0.113	1.57	0.050	0.418	0.750	0.211	0.423	0.655	0.770
Mesophiles	0.368	0.989	0.880	1.86	1.07	0.444	0.132	0.343	0.501	0.087	0.457
Molds/Yeast	0.809	0.997	0.754	6.25	5.05	0.417	0.285	0.243	0.176	0.536	0.233

## Data Availability

Study data are available on request from the corresponding author (L.E.R.-J).
